# Open-label placebo treatment to improve relaxation training effects in healthy psychology students: a randomized controlled trial

**DOI:** 10.1038/s41598-021-92579-0

**Published:** 2021-06-22

**Authors:** Anne Schienle, Isabella Unger

**Affiliations:** Department of Clinical Psychology, University of Graz, Universitätsplatz 2/DG, 8010 Graz, Austria

**Keywords:** Human behaviour, Signs and symptoms

## Abstract

Placebos, that are administered with deception, can reduce stress and increase relaxation. The present study investigated an open-label placebo (OLP) to improve the effects of Progressive Muscle Relaxation (PMR) training. A total of 160 psychology students were randomly assigned to a 14-day PMR course with or without daily OLP treatment. The placebo was administered along with an explanation of placebo effects and the verbal suggestion that the OLP activates the body’s natural relaxation response. The relaxation instructions for home practice were delivered via a smartphone app, which was also used for the evaluation of the exercises. The participants of the OLP group completed more PMR exercises (M = 9.75) than the group without a placebo (M = 8.15). The two groups did not differ in reported exercise-related changes in relaxation level. On average, the OLP group rated the effects of the placebo as very low and was characterized by a higher drop-out rate compared to the group without OLP. Some participants experienced the OLP as negative. In conclusion, factors that influence the acceptance of OLP treatment require further investigation.

## Introduction

Inert substances and sham interventions (placebos) can elicit beneficial effects on various health-related outcomes^1^. The positive placebo responses can be induced by diverse interrelated processes, such as behavioral conditioning (e.g., prior experiences of a treatment benefit), expectation (of a treatment benefit), and social cognition (e.g., the context of the treatment). The resulting placebo effects are mediated by multiple brain systems and neurochemical modulators. For example, one of the most studied placebo phenomena, placebo analgesia, is associated with activity changes in the pain modulatory network of the brain and the release of endogenous opioids^1,2^.

In standard placebo treatment, the recipients are not informed about the use of the placebo. The placebo providers create the deceptive impression of a 'real' treatment because this was viewed as a prerequisite for the placebo effect to occur. Despite the positive effects of ‘deceptive placebos’ in therapy and research, their use violates the ethical principles of respect for the autonomy of patients and study participants^3^. Alternative strategies are therefore required.

The ethical issue of deceptive placebo treatment can be circumvented by providing open-label placebos (OLPs). During OLP treatment the placebo recipients are fully informed that they receive an inert substance or intervention that is not known to directly cause an effect on a certain outcome. Different potential mechanisms of OLP effects have been discussed in the literature, including ‘pharmacological memory’ (OLPs act as conditioned cues that elicit previously learned responses) and ‘conscious expectancy’ (OLPs are often administered along with explicit positive suggestions which instill hope of improvement)^3–5^. A new theory ‘embodied cognition’ proposes that the physical action associated with the OLP treatment (e.g. taking the pill) is the key active OLP component. It is the behavior that subsequently leads to cognitions, which, in turn, induce the brain to produce a placebo response^4^.

Several studies have shown that OLPs can alleviate stress and anxiety in healthy individuals^6–9^. For example, El Brihi et al.^6^ assigned undergraduate students randomly to an OLP group or a no-treatment group. The OLP group was instructed to take one or four placebo pills (labeled as ‘Plaxibax’) over five days. Placebo-treated participants reported enhanced physical and psychological well-being (e.g., reduced emotional distress) independent of the placebo dose. In a large study with electroencephalography^7^, OLP treatment (with a nasal spray) reduced self-report and neural measures of emotional distress during the viewing of unpleasant pictures. In a pilot study^8^, a 2-week OLP treatment (two pills/day) reduced self-reports of test anxiety in university students. Kleine-Borgmann et al.^9^ assessed subjective well-being (e.g., stress) in healthy students during midterm exams as well as objective measures of test performance. The 21-day OLP application (two capsules/per day labeled ‘Zeebo’) improved well-being but did not affect overall test performance.

The mentioned studies used the OLP as the primary intervention. However, placebos can also be administered as an addition to another treatment (as adjunctive therapy). Here, the OLP is combined with a standard treatment (treatment as usual; TAU) to improve TAU (an increment in the degree of change generated by TAU). This approach has been applied in studies that tested the effects of Progressive Muscle Relaxation (PMR)^10,11^. PMR is based upon the simple practice of tensing one muscle group at a time followed by a relaxation phase with the release of tension. The reduction of muscle tensions leads to mental relaxation, however only with regular practice^10^. Compliance problems to practice PMR (and other relaxation techniques) are a common issue in clinical practice^12^. Often the time practiced is not sufficient to induce meaningful increases in relaxation levels.

The adherence to relaxation training can be positively influenced with placebo treatment. In a randomized controlled trial^10^, patients with major depressive disorder either performed daily relaxation exercises as therapeutic homework with a ‘deceptive placebo’ (DP introduced as herbal medicine that mobilizes the natural relaxation response of the body) or without the placebo. The placebo treatment increased the frequency and quality of the relaxation exercises. The patients in the placebo group practiced more often and reported greater relaxation effects. In a study with healthy students^11^, the DP was administered concurrently with PMR training. The participants were randomly assigned to a TAU group, which was instructed to practice PMR every day for two weeks, or a PMR + placebo group, which received additional daily placebo treatment. The placebo group practiced more often than TAU.

The present investigation examined whether an OLP can have similar effects as a DP used in previous relaxation studies^10,11^. The participants received the same placebo concerning the appearance, and dosage—except for a non-deceptive placebo administration. Psychology students were randomly assigned to a 14-day PMR course with or without daily OLP treatment. The participants received the placebo (a small blue bottle filled with sunflower oil) along with an explanation of placebo effects and the verbal suggestion that the placebo activates the body’s natural relaxation response. The placebo was orally administered (three drops) before each daily PMR practice. The relaxation instructions were delivered via a smartphone app, which was also used for the evaluation of the exercises.

It was hypothesized that the OLP would increase the frequency of relaxation practice, and the quality of practice (i.e. higher relaxation level in the OLP group compared to TAU). As an exploratory research question, we investigated whether the OLP would influence the reported stress/recovery level after the PMR course.

## Results

### Exploratory analyses

- Drop-out analysis: Twelve participants who had been assigned to the OLP group (15%) did not take the placebo. In the TAU group, there was no drop-out.
- Perceived effectiveness of the placebo: The effectiveness rating for the OLP was *M* = 2.22 (*SD* = 1.6, observed range 1–8; 1 = ‘very low’). Of the OLP participants, 44% gave a rating of ‘1’ and 25% of ‘2’. The OLP effectiveness rating correlated with the perceived effectiveness of PMR (*r* = 0.40, *p* = 0.001) and the attitude towards alternative/complementary medicine (*r* = 0.27, *p* = 0.029). Moreover, the PMR-induced increase in reported relaxation and pleasantness, and the decrease in arousal (post minus pre exercise) correlated with the perceived OLP effectiveness (relaxation: *r* = 0.38*, p* = 0.002; valence *r* = 0.33 , p = 0.007, arousal *r* = − 0.31; *p* = 0.013).

### Main analyses

**Figure 1:**
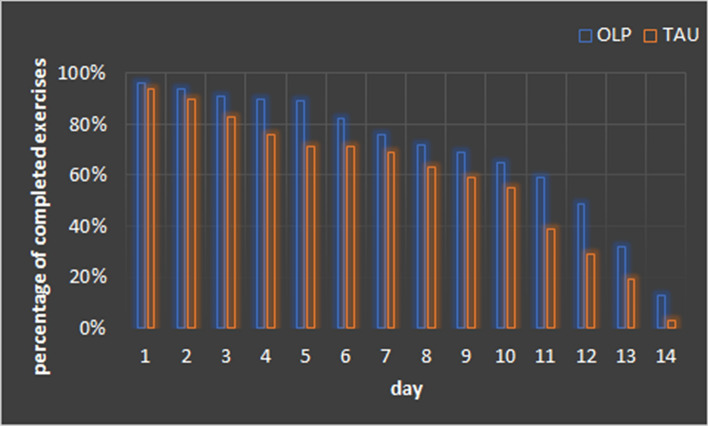
Percentage of completed Progressive Muscle Relaxation exercises per day and group. *OLP* open-label placebo, *TAU* treatment as usual without placebo.

- Exercise quantity
  The OLP group (*M* = 9.75, *SD* = 4.1) completed more relaxation exercises over the 14-day training period than the TAU group (*M* = 8.15, SD = 4.4; *t*(146) = 2.28, *p* = 0.024, Cohen’s *d* = 0.38). The percentage of completed daily PMR exercises per group ranged between 96% (day 1) and 13% (day 14) for the OLP group, and between 94% (day 1) and 3% (day 14) for the TAU group (see Fig. 1).
- Exercise quality
  Relaxation: The main effect Time reached statistical significance (*F*(1,138) = 325.84, *p* < 0.001, *ηp*^*2*^ = 0.70). Participants reported a higher relaxation level after the daily PMR exercise (see Table 1). The main effect Group and the interaction Group x Time were not significant (*p* > 0.71).
  Arousal: The main effect Time was significant (*F*(1,138) = 109.57, *p* < 0.001, *ηp*^*2*^ = 0.44). Participants reported a lower level of arousal after the daily PMR exercise. The other effects were not significant (*p* > 0.58).
  Valence: The main effect Time reached statistical significance (*F*(1,138) = 162.89, *p* < 0.001, *ηp*^*2*^ = 0.54). Participants reported a more positive affective state after the PMR exercise. The main effect Group and the interaction Group × Time were not significant (*p* > 0.47).
  The two groups did not differ in the perceived effectiveness of the PMR training (OLP: *M* = 4.75, *SD* = 1.92; TAU: *M* = 4.65, *SD* = 1.85; (*t*(146) = 0.32, *p* = 0.75).
- Reported stress/ recovery level

**Table 1:** Affective ratings (means, standard deviations) before and after the daily relaxation exercise.

	OLP group		TAU group	
	Before	After	Before	After
Relaxation	5.31 (1.01)	6.60 (0.93)	5.26 (0.97)	6.54 (0.89)
Valence	5.94 (0.94)	6.63 (0.92)	5.82 (0.95)	6.54 (0.88)
Arousal	2.97 (1.34)	2.34 (1.10)	2.94 (1.34)	2.37 (1.12)

*OLP* open-label placebo, *TAU* treatment as usual (without placebo).

Stress level: The main effect for Session reached statistical significance (*F*(1,146) = 9.19, *p* = 0.003, *ηp*^*2*^ = 0.059). The participants reported a lower stress level after the course (*M* = 1.26, SD = 0.61) than before the course (*M* = 1.40, *SD* = 0.64). The main effect Group (*p* = 0.25) and the interaction Group × Session (*p* = 0.69) were not significant.

Recovery level: None of the effects reached statistical significance (*p* > 0.12; before: *M* = 3.07, *SD* = 0.94.; after: *M* = 3.16, *SD* = 0.83).

## Discussion

The present study tested whether an OLP can improve relaxation training effects. Compared to a standard group (with no additional placebo treatment), the OLP group practiced more often during the 14-day PMR course (on average 1.6 sessions). The effect size of this frequency difference was small (Cohen’s *d* = 0.38). The effect of deceptive placebo treatment in a PMR study with the same design^11^ was higher (comparison deceptive placebo vs. no-placebo; Cohen’s *d* = 0.56). No OLP effects were found for exercise quality. The PMR training increased levels of experienced relaxation and pleasantness and reduced arousal. Moreover, the PMR course reduced perceived stress as indicated by the stress/recovery questionnaire^13^. However, these effects were comparable in the OLP and TAU groups.

The OLP had been introduced as a means to activate the body’s natural relaxation response. This suggestion could have produced an immediate (direct) response (i.e., a higher relaxation level in the OLP group before the exercise), or could have motivated the OLP recipients to practice more, which in turn could have positively affected the relaxation level (indirect placebo effect). The data showed, that the OLP did not have a direct effect; both groups reported a comparable baseline level of relaxation (arousal) before the daily practice. The OLP also did not increase the practicing time sufficiently to enhance the relaxation level. During the PMR course, the OLP group practiced 16 min more on average than TAU.

In general, the perceived OLP effectiveness was rated as very low (M = 2.2 on a scale with possible values ranging between 1: low and 9: very high). Of the participants, 69% chose 1 or 2. Moreover, the OLP group was characterized by a higher drop-out rate than the TAU group. Fifteen percent of the participants assigned to the OLP group did not take the placebo. A similar finding has been reported before^9^. In this study on psychological well-being and test performance in students, 11% of the participants assigned to the OLP group did not take the placebo as instructed. This shows that OLPs are not an option for everyone. A certain mindset seems to be a prerequisite for OLP acceptance. In the present study, a higher effectiveness rating for the OLP was correlated with a more positive attitude to complementary/alternative medicine.

In summary, the present study found a modest effect of an OLP to improve compliance with relaxation training. This small effect is very likely associated with the treated sample, which consisted of healthy students with a low stress level at the beginning of the intervention. However, previous research has limitations that may have led to an overestimation of OLP effects. For example, although the concept of OLPs explicitly refers to an open label, some authors^6,9^ used a brand name for the placebo (‘Plaxibax’, ‘Zeebo’). A brand name suggests a real substance. Other authors^13,14^ have advised caution when interpreting OLP results due to a lack of blinding in some studies, the fact that positive messages were included alongside OLPs, and that instructions often involved equivocation over how placebos work^14,15^. Therefore, study participants could easily be confused whether they indeed received a placebo. For example, in an OLP investigation on sports performance^16^, some participants questioned whether they had received a placebo (‘but I also ended up thinking that maybe it wasn’t a placebo’). Other participants of this study^16^ stated that they believed their performance worsened because they were told that the drug was a placebo. In the present study, commentaries about the OLP treatment also indicated that some participants perceived the OLP approach as negative (‘made me feel foolish’; ‘I felt annoyed to have to take the placebo’).

A study^17^ that investigated outcome expectations and general acceptance of OLPs found that the application of deceptive placebos (DP) was rated as more acceptable and outcome expectations were higher for DPs compared to OLPs. Because positive treatment attitudes are a central mechanism for psychological interventions that predict therapy success, future research should investigate the role of attitudes to OLPs in more detail.

Some limitations of the present study need to be taken into account. We recruited a sample of psychology students. Therefore, the results cannot be generalized to other groups. In the present experiment, we used a placebo oil instead of placebo pills as in other OLP studies^6,8,9^. It has been argued that meaningfully large placebo effects require a mixture of both prior experiences of treatment benefits and positive expectations^1^. OLP pills might be more effective to elicit previously learned responses (‘pharmacological memory’) due to their wider usage than oil. Moreover, we tested a sample of participants, who reported a relatively low stress level before the PMR course. Other OLP studies^9^ administered the placebo in times of stress (e.g., midterm exams) to allow placebo-associated improvement. Therefore, effects might be larger in individuals with a greater need for relaxation (e.g. patients with mental disorders, healthy individuals with an elevated stress level).

Positive aspects of this preregistered study include the large sample size, the unambiguous OLP approach (e.g., the bottle filled with the inert oil had the label ‘placebo’), and the fact that the experimenter was blinded concerning the group assignment. Thus, expectations of the placebo provider could not influence experiment outcomes.

## Conclusion

This study found a small OLP effect on the exercise quantity of PMR and no effect on exercise quality and perceived stress level. Factors that influence the acceptance of OLP treatment require further investigation.

## Method

The present study was approved by the ethics committee of the University of Graz, Austria (GZ 39/92/63 ex 2019/20) and carried out following the Declaration of Helsinki. Each participant provided informed consent. This study was preregistered on the open science framework (OSF). The study used a single-blinded approach; the experimenter did not know who received the OLP.

### Participants

**Figure 2:**
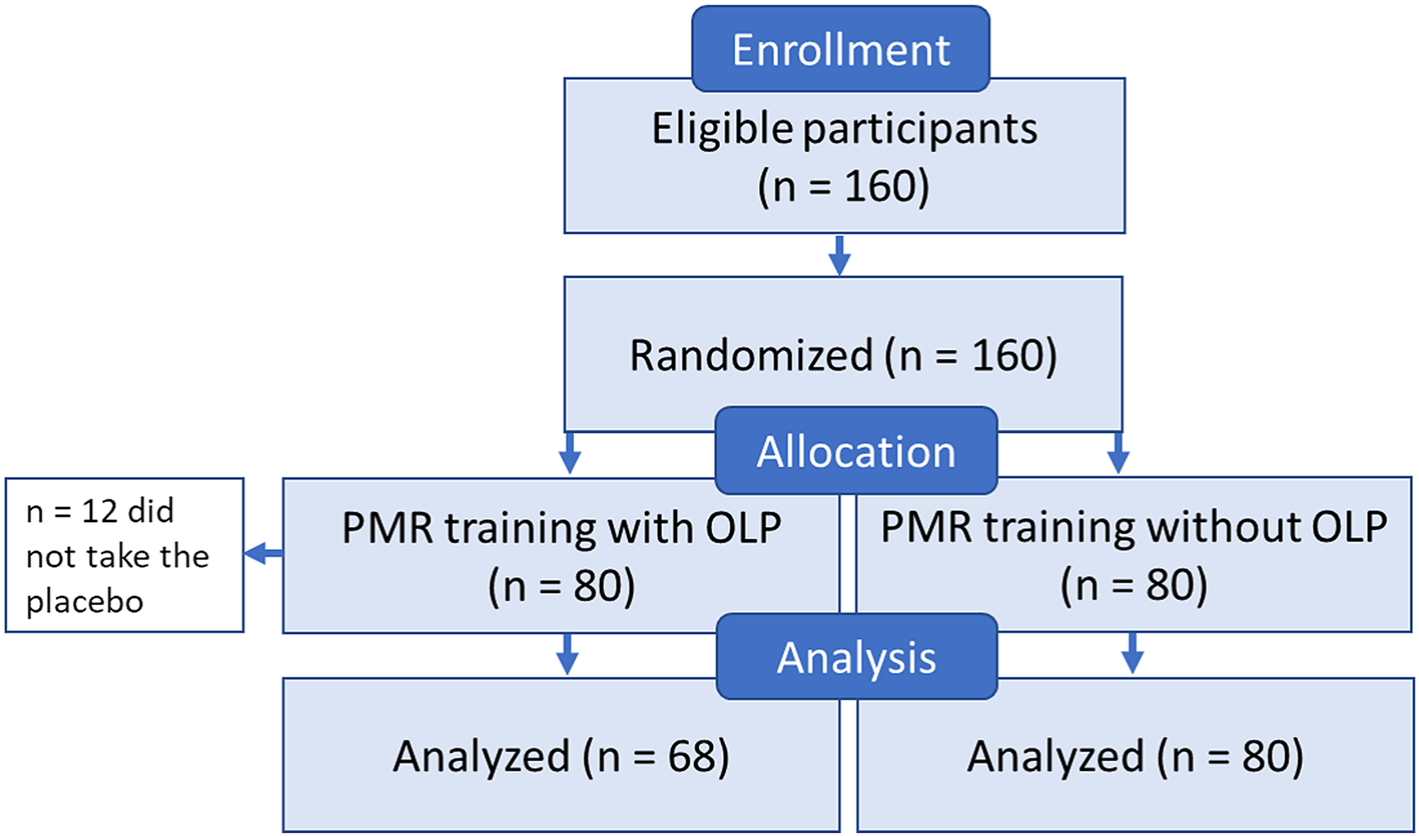
Consort flow diagram. *PMR* progressive muscle relaxation, *OLP* open-label placebo.

A total of 160 University students of a master’s psychology program participated in a 14-day PMR course. They were either assigned to a group with standard PMR training (treatment as usual; TAU; n = 80) or a PMR group with additional daily OLP treatment (OLP; n = 80). The sample size of the OLP group was reduced to n = 68 because 12 participants did not take the placebo (see Fig. 2). Thus, 148 students (71% female, 29% male; mean age = 24.4 years, SD = 2.7) comprised the final sample. The two groups (OLP, TAU) did not differ in mean age (t(146) = 1.29, p = 0.20), their attitude toward relaxation training (‘Do you believe that relaxation techniques have positive/health-promoting effects?’ 0–6; 6 = strong belief; M_OLP_ = 4.59, SD = 1.12; M_TAU_ = 4.74, SD = 0.99; t(146) = 0.85, p = 0.39) and alternative/complementary medicine (‘Do you believe that complementary/alternative medicine has positive/health-promoting effects?’ 0–6; 6 = strong belief; M_OLP_ = 3.01, SD = 1.75; M_TAU_ = 3.15, SD = 1.70, t(146) = 0.47, p = 0.64). Of the participants, 22% reported previous experience with relaxation techniques (the percentage did not differ between OLP and TAU).

### Experimental procedure

The participants were randomly assigned (with a random number table) to the OLP or TAU group. All participants conducted a brief PMR training^18^. They were instructed how to tense and relax four different muscle groups (arms, shoulders/neck, face, legs; according to a brief PMR training). The guided PMR exercise lasted 10 min. A smartphone app was used for the delivery of the PMR instructions, the monitoring of adherence to home practice, and the evaluation of the exercises by the participants (e.g., perceived changes in relaxation level). The participants were asked to complete one training session per day. They were free to choose the time of the day for the session, so it would fit in their daily schedule. (If a participant would have decided to practice more than once per day, this would have been registered by the app. This was however not the case).

The OLP participants received 30 ml sunflower oil provided in a blue glass bottle with a dropper for oral administration. The label of the bottle stated ‘placebo’. The participants were instructed to take 3 drops of the oil per day, directly before each PMR session. It was stated that the placebo would support the body’s natural relaxation response. Moreover, the participants received information about placebos in a video that followed the instruction as described in the study by Kaptchuk^18,19^. It was stated that placebos made of an inert substance (like sugar pills) have been shown in scientific studies to produce significant improvement in various conditions (e.g., reduction of negative emotions, stress) through a mind–body self-healing process. The participants were further informed that the placebo effect is powerful, the body can learn to respond to placebos through conditioning, positive expectations/beliefs are helpful but not necessary, and that taking the placebo faithfully is crucial. At the end of the 14-day training, the participants of the OLP group were asked to rate the perceived effectiveness of the OLP (1 = not effective; 9 = very effective). Both groups rated the perceived effectiveness of the PMR training (1–9; 9 = very effective). The participants of the OLP group were also asked to bring the bottles back at the end of the study period. A drop-off box was provided to allow an anonymous return of the bottles. Six participants returned a full bottle; another six participants returned no bottle but wrote a commentary that they did not take the placebo.

Before and after the PMR course, the participants completed the Recovery-Stress Questionnaire^13^ (RSQ). The RSQ assesses perceived stress and the ability to recover from stressful situations. We computed a total stress score and a total recovery score for each participant (recovery scale: α = 0.95; stress scale: α = 0.93). Possible mean scores range between 0 and 6 with higher scores indicating higher levels of stress and recovery.

### Data acquisition

The data gathering (conducted over 14 days) was achieved by combining a PWA (Progressive Web App) and a remote server for storage. The server was encrypted through an SSL connection. Participants first installed the PWA on their mobile phones and then answered three questions concerning their current level of relaxation, pleasantness, and arousal using nine-point Likert scales (1–9; 9 = very pleasant, aroused, relaxed). After listening to the PMR audio file, the participants repeated the rating. The survey was conducted via a webpage created with HTML, CSS, and Javascript (using the Vue.js Framework). The anonymous data were sent to a remote server where a Python Flask script handled the data collection and created a CSV file for each participant.

### Statistical analysis

To test the effects of Group (TAU, OLP) on relaxation quantity (number of completed relaxation exercises) we computed a t-test. A mixed model analysis of variance (ANOVA) was performed to test the effect of Group and Time (before/after relaxation exercise) on the reported level of relaxation/arousal/pleasantness. An additional ANOVA tested the effect of Session (before/after relaxation course) and Group on the perceived level of stress and recovery (RSQ scores). We report ηp^2^ (partial eta squared) as an effect size measure. Exploratory correlation analyses were conducted for the OLP group to test the association between the perceived effectiveness of the placebo and the quantity/ quality of the relaxation exercises.

## Data Availability

The dataset generated during and/or analyzed during the current study is available from the corresponding author on reasonable request.
